# miR-15a/miR-16 down-regulates BMI1, impacting Ub-H2A mediated DNA repair and breast cancer cell sensitivity to doxorubicin

**DOI:** 10.1038/s41598-017-02800-2

**Published:** 2017-06-27

**Authors:** Nibedita Patel, Koteswara Rao Garikapati, Raj K. Pandita, Dharmendra Kumar Singh, Tej K. Pandita, Utpal Bhadra, Manika Pal Bhadra

**Affiliations:** 1 Centre for Chemical Biology, CSIR-Indian Institute of Chemical Technology, Tarnaka, Hyderabad, Telangana State 500007 India; 2 Gene Silencing Group, Centre for Cellular and Molecular Biology, Hyderabad, Telangana State 500007 India; 3 Academy of Scientific and Innovative Research (AcSIR), Training and Development Complex, CSIR Campus, CSIR Road, Taramani, Chennai 600 113 India; 4 Department of Radiation Oncology, Weill Cornell Medical College, The Methodist Hospital Research Institute, Houston, TX 77030 USA

## Abstract

The B-lymphoma Moloney murine leukemia virus insertion region-1 protein (BMI1) acts as an oncogene in various cancers, including breast cancer. Recent evidence suggests that BMI1 is rapidly recruited to sites of DNA double strand breaks where it facilitates histone H2A ubiquitination and DNA double strand break repair by homologous recombination. Here we show that miR-15a and miR-16 expressionis decreased during the initial period after DNA damage where it would otherwise down-regulate BMI1, impairing DNA repair. Elevated miR-15a and miR-16 levels down-regulated BMI1 and other polycomb group proteins like RING1A, RING1B, EZH2 and also altered the expression of proteins associated with the BMI1 dependent ubiquitination pathway. Antagonizing the expression of miR-15a and miR-16, enhanced BMI1 protein levels and increased DNA repair. Further, overexpression of miR-15a and miR-16 sensitized breast cancer cells to DNA damage induced by the chemotherapeutic drug doxorubicin. Our results suggest that miR-15a and miR-16 mediate the down-regulation of BMI1, which impedes DNA repair while elevated levels can sensitize breast cancer cells to doxorubicin leading to apoptotic cell death. This data identifies a new target for manipulating DNA damage response that could impact the development of improved therapeutics for breast cancer.

## Introduction

The BMI1 (B cell-specific Molony murine leukemia virus integration site (1) is a componentof the polycomb repressive complex (PRC1) that stimulates the E3 ubiquitin ligase activity of PRC1 via binding to the catalytic subunit RING2/RING1b^1^. BMI1 is a transcriptional repressor, which plays an important role in self-renewal and differentiation of stem cells^2–4^. BMI1 also represses the expression of p16, which induces cellular senescence and cell death^5,6^. Overexpression of BMI1 has been identified in various cancer tissues^7–9^ and in breast cancer it is associated with poor prognosis, contributing to cell proliferation, invasion, and metastasis^10,11^. Several approaches have been examined in an effort to develop cancer therapeutics targeting BMI1^12–15^, particularly since BMI1 has a significant role in DNA damage response pathway^16–19^. Loss of BMI1 impedes DNA double-strand break repair by homologous recombination thereby increasing radiosensitivity. It was found that BMI1 rapidly assembles at sites of DNA damage and mono-ubiquitinates histone H2A at lysine (K)119(H2A-K119), γ-H2AX to induce DNA repair^19–24^. This activates several signalling pathways and modifies the chromatin structure for subsequent association of DNA repair proteins. BMI1 is involved in DNA double strand break repair by facilitating the phosphorylation of H2AX by ATM, and the recruitment of ATR, E3-ubiquitin ligase RNF8, RNF168, BRCA1, Abraxas and 53BP1 to the site of DNA damage^25,26^ to produce homology-dependent DNA double strand break repair.

MicroRNAs (miRNA) are small non-coding regulatory RNA molecules (22 nucleotides in length) involved in diverse biological processes^27–29^. microRNAs negatively regulate gene expression at the post-transcriptional level by binding to complementary sequences in the coding 3′ untranslated region of their target messenger RNA(mRNA)^30–32^. A single miRNA may repress multiple different transcripts, pathways and responses by altering protein expression, or several miRNAs may control a single pathway^33^. microRNAs have been shown to regulate DNA repair factors and oncogenes. For example, the 3′UTR of ATM mRNA is targeted by miR-421, miR-100, and miR-18a to down-regulate its protein expression^34–36^. Similarly, ATR is targeted by miR-185^37^, MDM2 is targeted by miR-25, miR-32, miR-18b and miR-661^38–40^ while BCL2 is targeted by miR-34a^41^.

In the present study, we demonstrate that miR-15a and miR-16 target BMI1. Ectopic expression of miR-15a or miR-16, or both impaired the DNA damage response to etoposide-induced DNA damage. Results from the reporter assay of BMI1 3′UTR as well as levels of BMI1 protein expression upon ectopic expression of miR-15a, miR-16 or both showed a significant decrease, whereas inhibition of endogenous levels of miR-15a, mir-16 along with overexpression of BMI1 reversed the effect and resulted in the regain of DNA repair response that facilitated cell survival. We observed that in etoposide-induced DNA damage response, ectopic expression of miR-15a, miR-16 induced up-regulation of the phosphorylation of DNA damage related proteins like γ-H2AX, p-CHK2, p-ATM, p53BP and down-regulation of BMI1, RING1A, RING1B, ub-H2A, RNF8, RNF168, MEL18 and BRCA1. In the present study for the first time, we showed a significant role of miR-15a and miR-16 in DNA damage repair by targeting BMI1. Also, interestingly, overexpressed miR-15a, miR-16 not only suppressed BMI1 level but also sensitizes breast cancer to chemotherapeutic drug doxorubicin by triggering intrinsic apoptosis in breast cancer cells. Therefore, we have shown the role of specific miRNAs involved in regulating the expression of BMI1 in response to DNA damage and BMI1 dependent ubiquitination pathway in breast cancer cells.

## Results

### miR-15a/16 levels are decreased during etoposide induced DNA damage response

**Figure 1:**
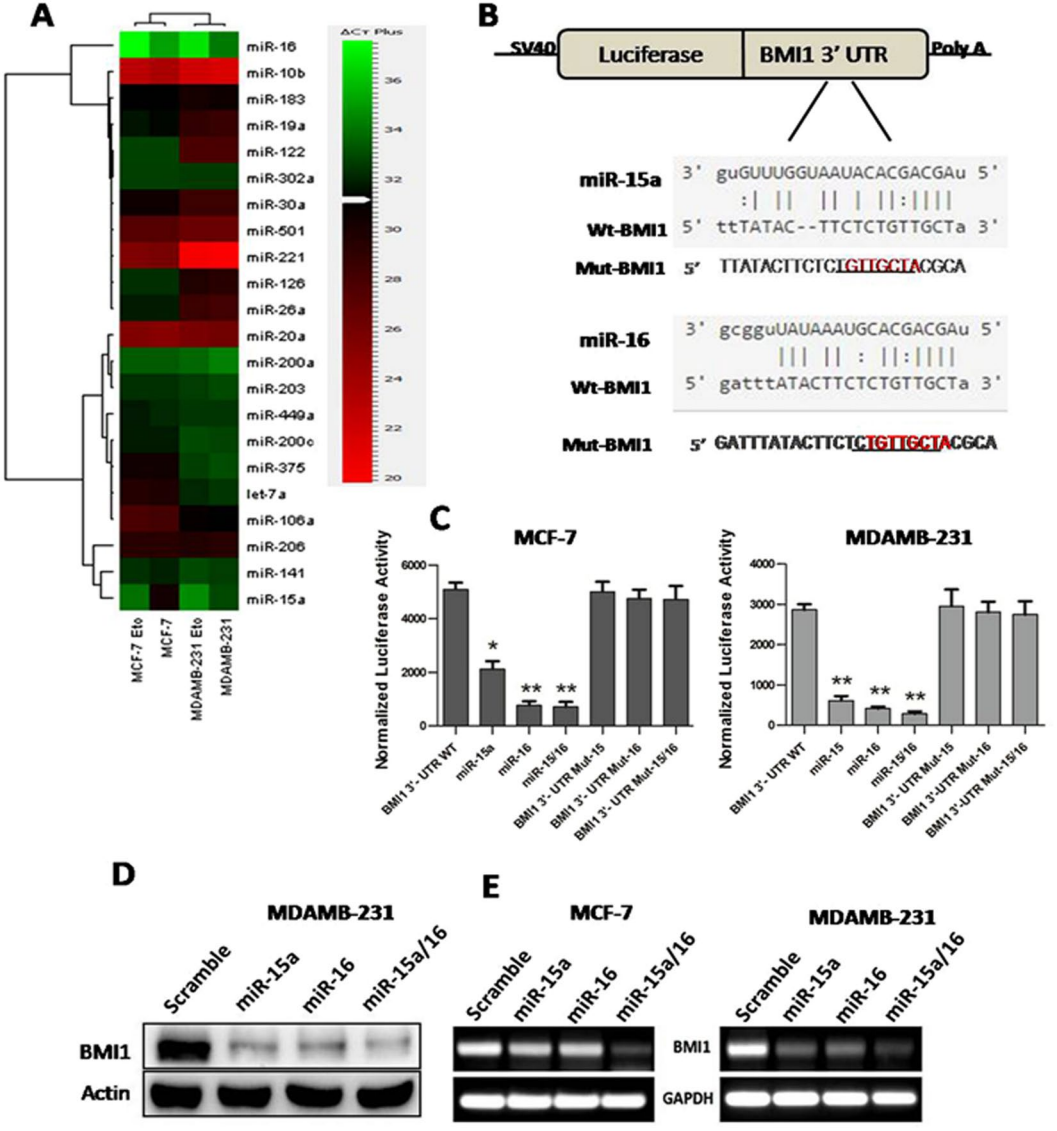
miR-15a/16 expression is altered in chemically induced DNA damage and it suppresses the BMI1 expression by targeting 3′UTR. Heat map showing the endogenous expression of miRNAs. The Green colour in the heat map indicates decreased and red colour indicates increased mRNA expression level. U6 was used as endogenous control (**A**). Predicted miRNA sequences and their recognition sites within 3′UTR of BMI1 (**B**) Renilla luciferase reporter assay showing the reporter expression in MCF-7 and MDAMB-231 cells co-transfected with wild-type 3′UTR of BMI1and mutant 3′UTR of BMI1 along with miR-15a, miR-16. Scramble miRNA vector used as a control. Renilla luciferase activity was normalized to firefly luciferase. n = 3, Students t-test was used to generate P values, *, ** and*** indicates P value summary (**C,D**). Western blot and RT-PCR data showing the expression of BMI1 upon overexpressed miR-15a, miR-16 and both. Blots were cropped to enhance the representation (E).

In order to identify miRNAs involved in the DNA damage response (DDR) and in modulating DNA repair gene expression, we created a DNA damage response model using etoposide, a topoisomerase-II inhibitor extensivelyused in DNA damage studies^42,43^. Continuous etoposide exposure induces persistent DNA damage but DNA repair^43^ is initiated after its withdrawal. Our dose-dependent study showed 50% cell death at 5 µM concentration (Fig. S1) where it efficiently induced significant DNA damage and by changing the media of the etoposide-treated cells, the cells were able to repair the damage to a certain extent (Fig. S2), therefore, we restricted our further studies to this concentration. MCF-7 and MDAMB-231 cells were grown and treated with etoposide (5 μM) for 2 hrs. The RNA was isolated and miRNA expression was quantified by real-time PCR using miRNAs human q-PCR primers (System biosciences Cat #. RA660A). Both miR-15a and miR-16 levels were decreased in etoposide-treated samples whereas most of the other 21miRNAs surveyed were unchanged (Fig. 1A). Using the miRNA binding site prediction tool miRTarBase (http://mirtarbase.mbc.nctu.edu.tw/), we identified miR-15a and miR-16 as potentially binding to the BMI13′UTR. To confirm miRNA binding, we cloned the wild-type and mutated 3′UTR of BMI1, into the psiCHECK-2 vector, a dual luciferase reporter system, and co-transfected the vectors along with a lentiviral vector expressing miR-15a, miR-16 into MCF-7 and MDAMB-231 cells. Cells transfected with empty psiCHECK-2 vector and scramble miRNA vector were used as controls. Luciferase reporter activity from an expression vector containing the wild-type BMI1 3′UTR was significantly decreased in both cell lines when co-transfected with miR-15a and miR-16 expression vectors. In contrast, reporter gene activity of a vector containing a Mut-3′ BMI1 3′UTR was largely unaffected by miR-15a and miR-16 expression (Fig. 1C). Moreover, overexpression of the miRNAsin MDAMB-231 cell line decreased endogenous BMI1 protein levels (Fig. 1D). miRNAs may affect mRNA degradation and translation. Overexpression of the miRNAs in MDAMB-231 shows significant down-regulation of mRNA expression of BMI1 but in MCF-7 show down-regulation of mRNA expression of BMI1 not as significant as MDAMB-231 (Fig. 1E). This dramatic etoposide-induced change in miR-15a and miR-16, which are predicted to target BMI1 expression, suggests their possible involvement in regulating the DDR.

### miR-15a/16 regulates repair of etoposide-induced DNA damage

**Figure 2:**
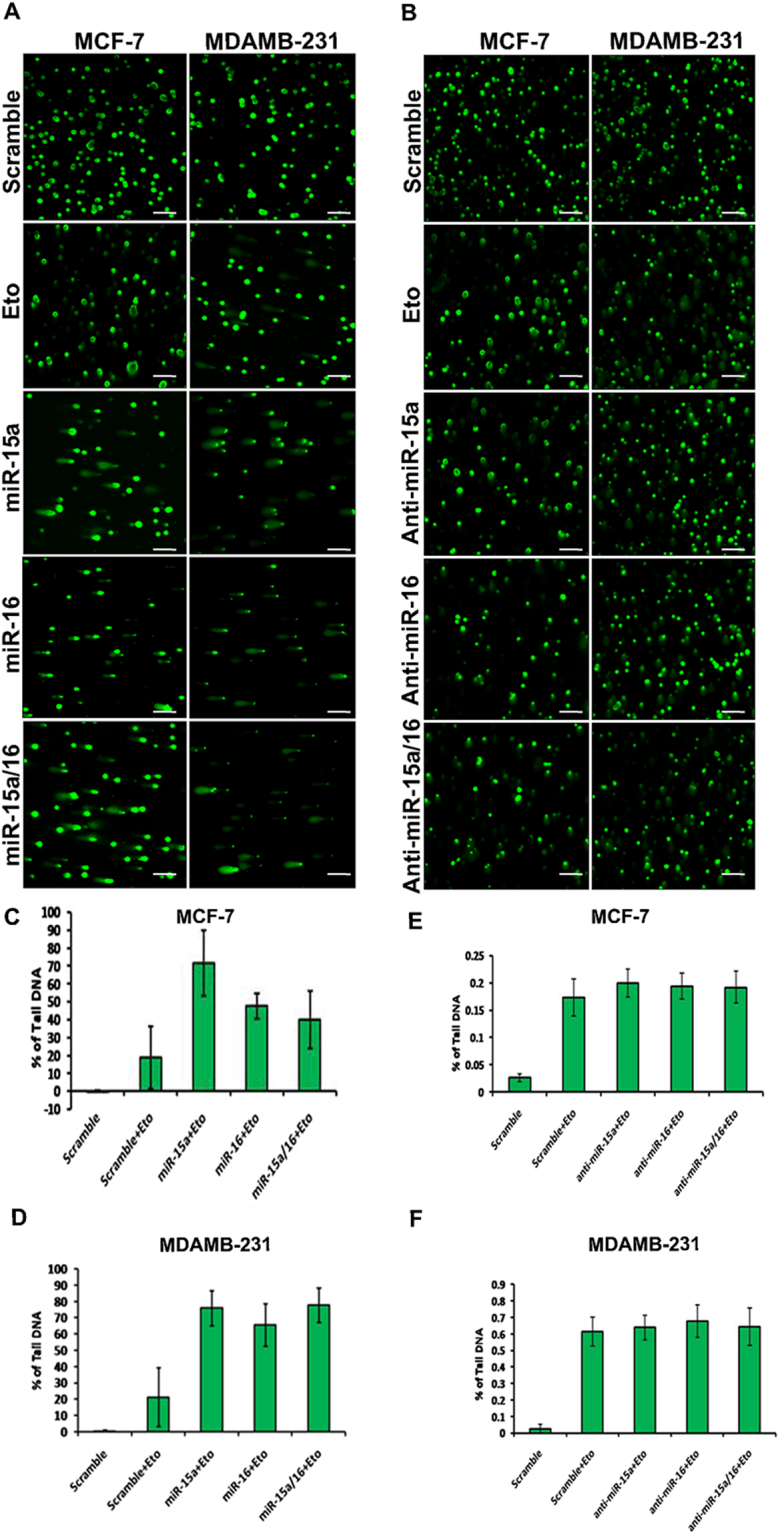
Ectopic expression of miR-15a/16 intensifies DNA damage and repair. Comet assay in MCF-7 and MDAMB-231 cells after transfection with miR-15a, miR-16, both miR-15a/16 and anti-miR-15a, anti-miR-16 and both. Etoposide was used to chemically induced DNA damage. Cells were processed for single-cell gel electrophoresis (comet assay) and miR-15a, miR-16 or both miR-15a/16 transfected cells show an increase in DNA damage as compared to cells transfected with scrambled miRNAs and anti-miRs under same damage condition. Bar indicates 150 µm (**A,B**). Cells with DNA damage that showed prominent tail (Comet) were scored using cometIV software and percentage of tail DNA was plotted (**C,D,E,F**).

To determine whether either miR-15a or miR-16 or both mediate DNA repair through BMI1, we performed single-cell gel electrophoresis (comet assay) of cells transfected with miR-15a, miR-16, both miR-15a/16, anti-miR-15a, anti-miR-16 and both. MCF-7 and MDAMB-231 breast cancer cells transfected witheither miRNAs or anti-miRs were incubated for 36 hrs followed by treatment with etoposide for 2 hrs than allowed torecover without drug for 8 hrs. A significant increase in the amount of DNA damage in cells transfected with miR-15a, miR-16 or both miR-15a/16 (Fig. 2A) was observed as evidenced by the prominent DNA tail (Comet), scored using cometIV software and plotted quantitatively to measure the induced DNA damage (Fig. 2C,D). Moreover, the cells transfected with anti-miRs did not produce asignificant number of comets as compared to the scramble miRNA vector transfected cells used as control (Fig. 2B,E,F). From this, we can conclude that miR-15a, miR-16 impair DNA repair and thus DNA damage is unrepaired in etoposide-treated breast cancer cells.

### miR-15a/16 modulates DNA repair through the BMI1-dependent ubiquitination pathway and alters the expression of associated proteins

**Figure 3:**
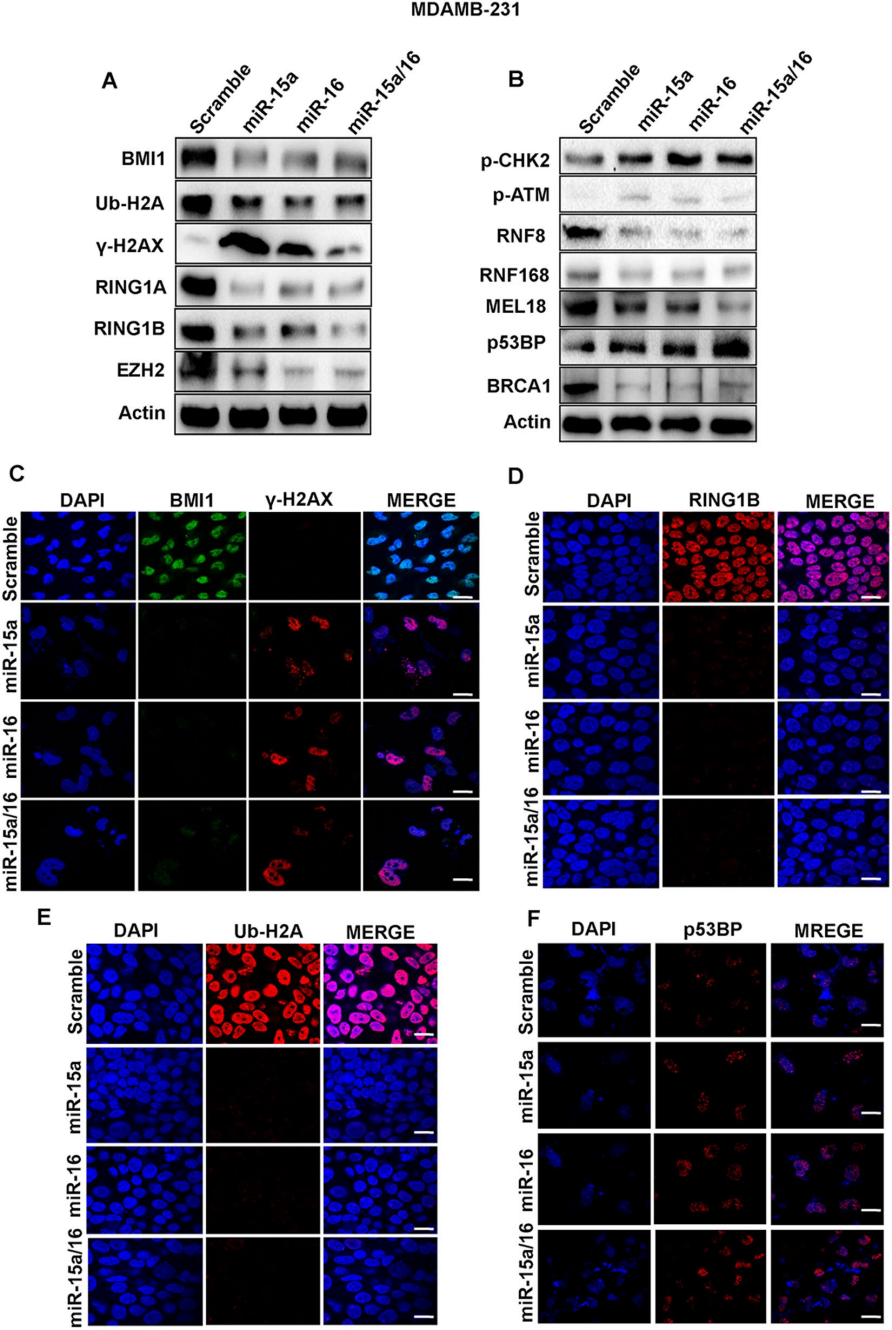
Ectopic expression of miR-15a, miR-16 sensitizes breast cancer cells to DNA damage through BMI1 dependent ubiquitination pathway. Western blot analysis showing the expression of BMI1, RING1A, RING1B, EZH2, γ-H2AX, Ub-H2A, p-CHK2, p-ATM, RNF8, RNF168, MEL18, p53BP, BRCA1 proteins in MDAMB-231 cells transfected with miR-15a, miR-16 or both miR-15a/16 under etoposide-induced DNA damage conditions.. Actin served as a gel loading control. Blots were cropped to enhance the representation (**A,B**). Immunofluorescence data showing accumulation of BMI1, ϒ-H2AX (**C**) RING1B (**D**) and Ub-H2A (**E**) p53BP (**F**) in MDAMB-231 cells transfected with miR-15a miR-16 or both miR-15a/16 under etoposide- treated conditions. Bar indicates 200 μm.

**Figure 4:**
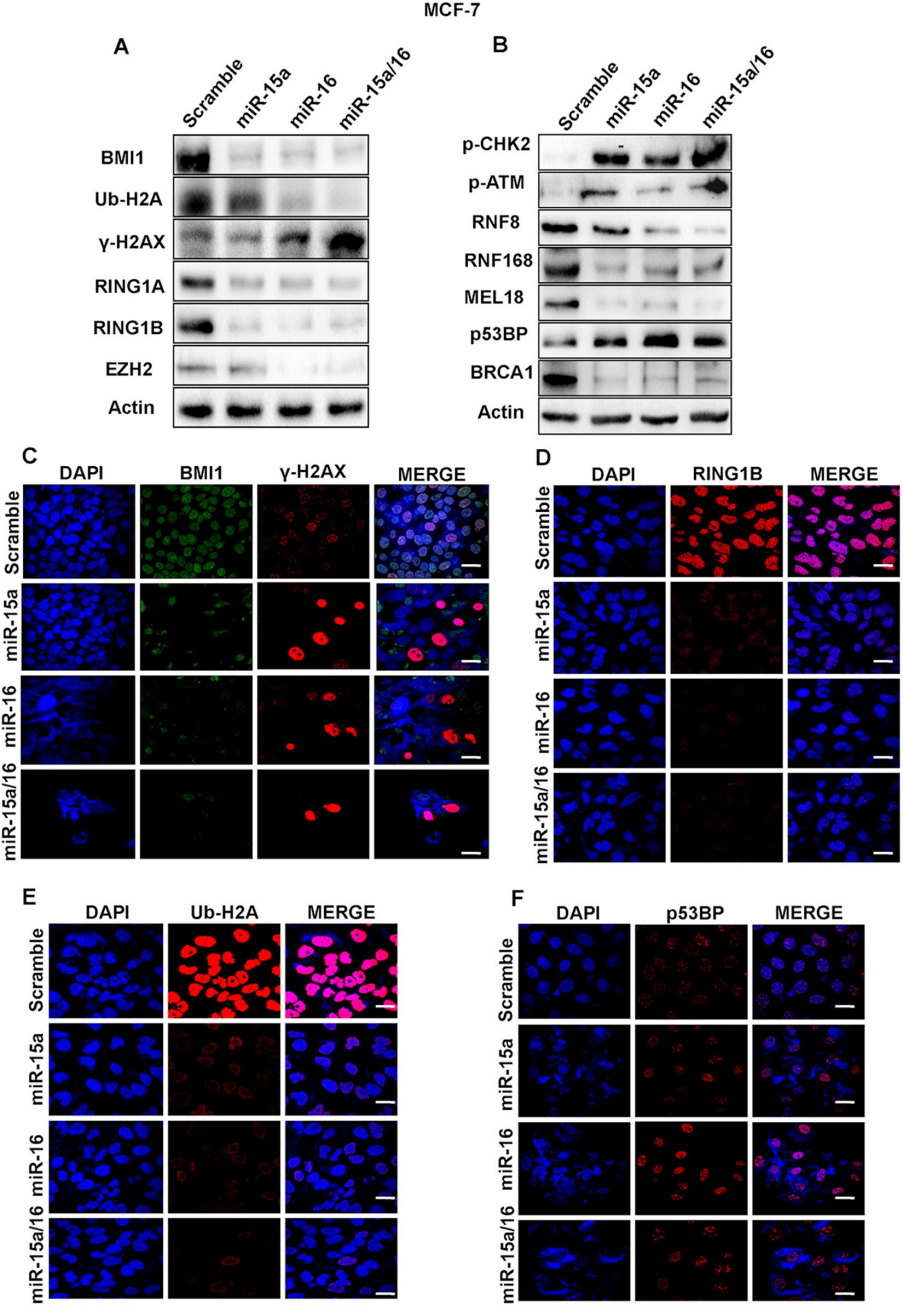
Western blot analysis showing the expression of BMI1, RING1A, RING1B, EZH2, γ-H2AX, Ub-H2A, p-CHK2, p-ATM, RNF8, RNF168, MEL18, p53BP, BRCA1 proteins in MDAMB-231cells transfected with miR-15a, miR-16 or both miR-15a/16 under etoposide-induced DNA damage conditions. Actin served as a gel loading control. Blots were cropped to enhance the representation (**A,B**). Immunofluorescence data showing accumulation of BMI1, ϒ-H2AX (**C**) RING1B (**D**) and Ub-H2A (**E**) p53BP (**F**) in MDAMB-231 cells transfected with miR-15a miR-16 or both miR-15a/16 under etoposide- treated conditions. Bar indicates 200 μm.

Our initial study confirmed that miR-15a and miR-16 regulate BMI1 expression by binding to its 3′UTR, and impair DNA repair. In order to better understand the underlying mechanism, we transfected both MCF-7 and MDAMB-231 cells with miR-15a and miR-16, both individually and in combination. Transfected cells were grown for 36 hrs, treated with etoposide for 2 hrs then allowed to recover in the absence of drug for 8 hrs. Nuclear protein extracts were prepared fromthe cells and analyzed for levels of BMI1 and associated polycomb group proteins (RING1A, RING1B, and EZH2) which play a crucial role in DDR and also promotion oftumorogenesis^24,44^. A significant BMI1down-regulation was seen in cells transfected with miR-15a, 16 or both as compared to control cells transfected with scrambled miRNAs. Protein levels of RING1A, RING1B, EZH2 were also significantly down-regulated under the same conditions (Figs 3A and 4A).

After DNA damage, BMI1 mono-ubiquitinates, histone H2A at K119 (H2A-K119ub), which is a critical step in initiating repair of damaged DNA^21^. We, therefore, determined the level of H2A-K119 ubin both MCF-7 and MDAMB-231 cell lines treated with etoposide and transfected with miR-15a, miR-16 or both by Western blot analysis. A significant down-regulation in the level of H2A-K119ub was observed compared to the control cells (Figs 3A and 4A). Under similar conditions, we measured the levels of γ-H2AX, amarker for DNAdouble-stranded breaks^45^, and found a significant increase in γ-H2AX levels as compared to control cells afteretoposide treatment (Figs 3A and 4A).

Protein extractsfrom cells ectopically expressing miR-15a, miR-16 in combination and separately, were also collected and analyzed by western blot analysis for levels of BMI1 dependent DNA damage and repair-related proteins p-CHK2, p-ATM, RNF8, RNF168, MEL18, p53BP, and BRCA1. The etoposide treated cells transfected with miR-15a, miR-16 or both showed a clear down-regulation of BRCA1, RNF8, RNF168, MEL18 levels along with up-regulation of p-ATM, p-CHK2, p53BPcompared to cells that underwent etoposide treatment but were transfected with scrambled miRNA. The results clearly demonstrated the role of miR-15a, miR-16 in DNA double strand break repair (Figs 3B and 4B).

To visualize the localization of BMI1, RING1A, RING1B, Ub-H2A, γ-H2AX, p-CHK2 and p53BP1at the cytological level, immunofluorescence studies were performed in both control and etoposidetreated cells with the corresponding antibodies. Cellstransfected with miR-15a, miR-16, and both miR-15a/16 showed less accumulation of BMI, RING1A, RING1B, Ub-H2A focialong with large accumulations of γ-H2AX and moderate accumulation of p-CHK2, p53BP1foci in MCF-7 and MDAMB-231 cells(Figs 3C,D,E,F and 4C,D,E,F) (Fig. S3A,B,C,D).

### Time-dependent expression of BMI1, γ-H2AX, Ub-H2A119 protein after ectopic expression of miR-15a/16

**Figure 5:**
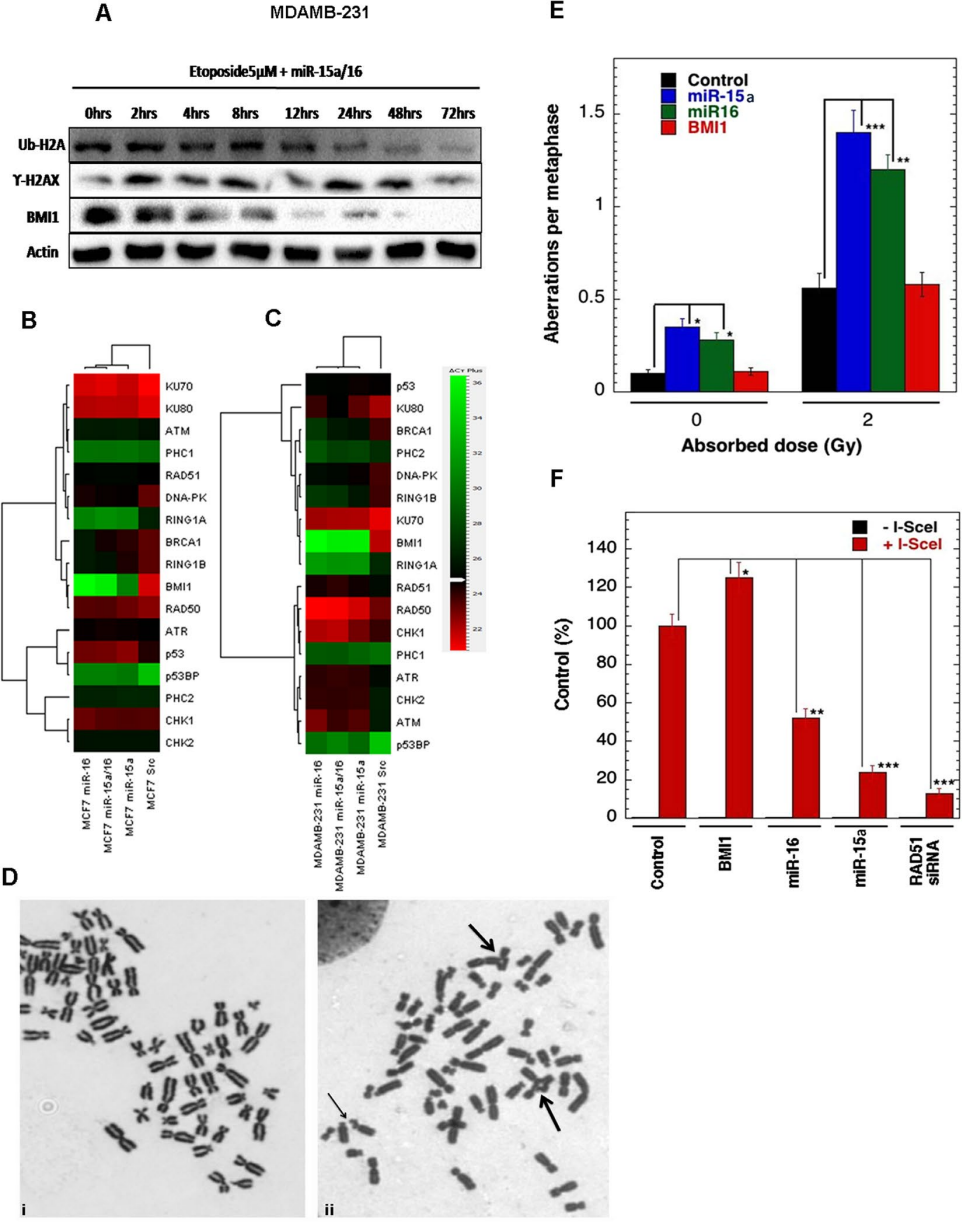
Time-dependent expression of proteins after ectopic expression of miR-15a/16 and overexpression of miR-15a/16 impedes homologous recombination-mediated repair. Western blot analysis showing the expression of BMI1, γ-H2AX, Ub-H2A in MDAMB-231 cells transfected with both miR-15a/16 followed by treatment with etoposide for 2 to 72 hrs. Actin was used as a gel loading control. During the course of time periods (0 hrs to 72 hrs). Blots were cropped to enhance the representation (**A**). Heat map showing the endogenous expression of quantitative RT-PCR analysis. The Green colour in the heat map indicates decreased and red colour indicates increased mRNA expression level. Gene specific primers were used for the amplification. GAPDH was used as endogenous control (**B,C**) Metaphase chromosomes of cells overexpressing Mir-15a/16. (i) Metaphase fromcontrol, (ii) Metaphase from irradiated cell showing gaps (small arrow) and radials (large arrows)in human H1299 cells (**D**). In human H1299 cells S-phase specific chromosome aberrations after 2 Gy irradiations. *p < 0.05, **p < 0.01, ***p < 0.001 Student *t*-test (**E**) Homologous recombination (HR) repair of DNA DSB repair in human H1299 cells are shown with and without I-SceI induction.*p < 0.05, **p < 0.01, ***p < 0.001Student *t*-test (**F**).

We next conducted time course studies for BMI1, γ-H2AX, Ub-H2A119 protein levels in cells transfected with miR-15a/16 and treated with etoposide for a period of 2-72 hrs. Time course studies also demonstrated down-regulation of BMI1, ubiquitination of H2A at lysine 119 and up-regulation of γ-H2AX. In all the experimental conditions, results were compared with etoposide-treated cells transfected with scramble miRNA vector that served as a control (Fig. 5A). These results strongly suggest that miR-15a, miR-16, and both miR-15a/16 have a strong impact in DNA damage response leading the cells towards apoptosis.

miRNAs can affect mRNA degradation and translation^46^. To check the expression levels of genes involved in DDR pathway that play important role in DNA damage and repair (BMI1, RING1A, RING1B, PHC1, PHC2, ATM, ATR, BRCA1, CHK1, CHK2, Tumor suppressor p53-binding protein1 (p53BP), p53, KU70, KU80, RAD50, RAD51, DNA-PK) upon miR-15a, miR-16, and both miR-15a/16 overexpression, we performed real-time PCR analysis with primers designed uniquely for the genes under analysis. There was a significant down-regulation of mRNA expression of BMI1 in cells transfected with overexpressed miR-15a, miR-16, and both miR-15a/16. A partial decrease in the mRNA expression of RING1A, RING1B, BRCA1, DNA-PK was also observed in both MCF-7 and MDAMB-231cells. ATM and ATR mRNA expressions were partially increased in MDAMB-231 cells, whereas the expression levels of other genes were unaltered in all the experiments. GAPDH was used as endogenous control (Fig. 5B,C).

### miR-15a/16 expression impacts HR-mediated repair

To determine whether overexpression of miR-15a/miR-16 and BMI1 has an impact on chromosome break repair, we examined cell cycle specific chromosomal aberrationsat the metaphase stage. Cell cycle stage-specific chromosome aberrations were analyzed at the metaphase after irradiation as previously described (Singh M, *et al*.^47^). As a result,no difference in G-1 or G-2 specific aberrations were found in cells with and without overexpression of miR-15a/miR-16, and BMI1, however, an increased number of S-phase specific aberrations (radials) was observed in cells overexpressing miR-15a/miR-16, suggesting that depletion of BMI1 does effect on the HR related repair as indicated by increased number ofS-phase specific chromosomal aberrations (Fig. 5D,E)

Double strand break repair follows two major pathways; homologous recombination (HR) and non-homologous end-joining (NHEJ)^48^. BMI1contributesto DNA double-stranded break repair by homologous recombination^25^. To determine whether miR-15a, miR-16 impact HR repair, we performed homologous recombination-assayby using I-Scel induced DNA DSB in green fluorescent protein (GFP) reconstitution assays. Induction of a site specific DSB was performed as described previously (Gupta A *et al*.^49^). As a result,we found that thecells overexpressing miR-15a/miR-16 has reduced HR mediated DSB repair, whereas the cells overexpressing BMI1 have increased frequency of HR mediated DSB repair (Fig. 5F).

### miR-15a/16 mediate suppression of BMI1 and induce G2/M checkpointactivation

**Figure 6:**
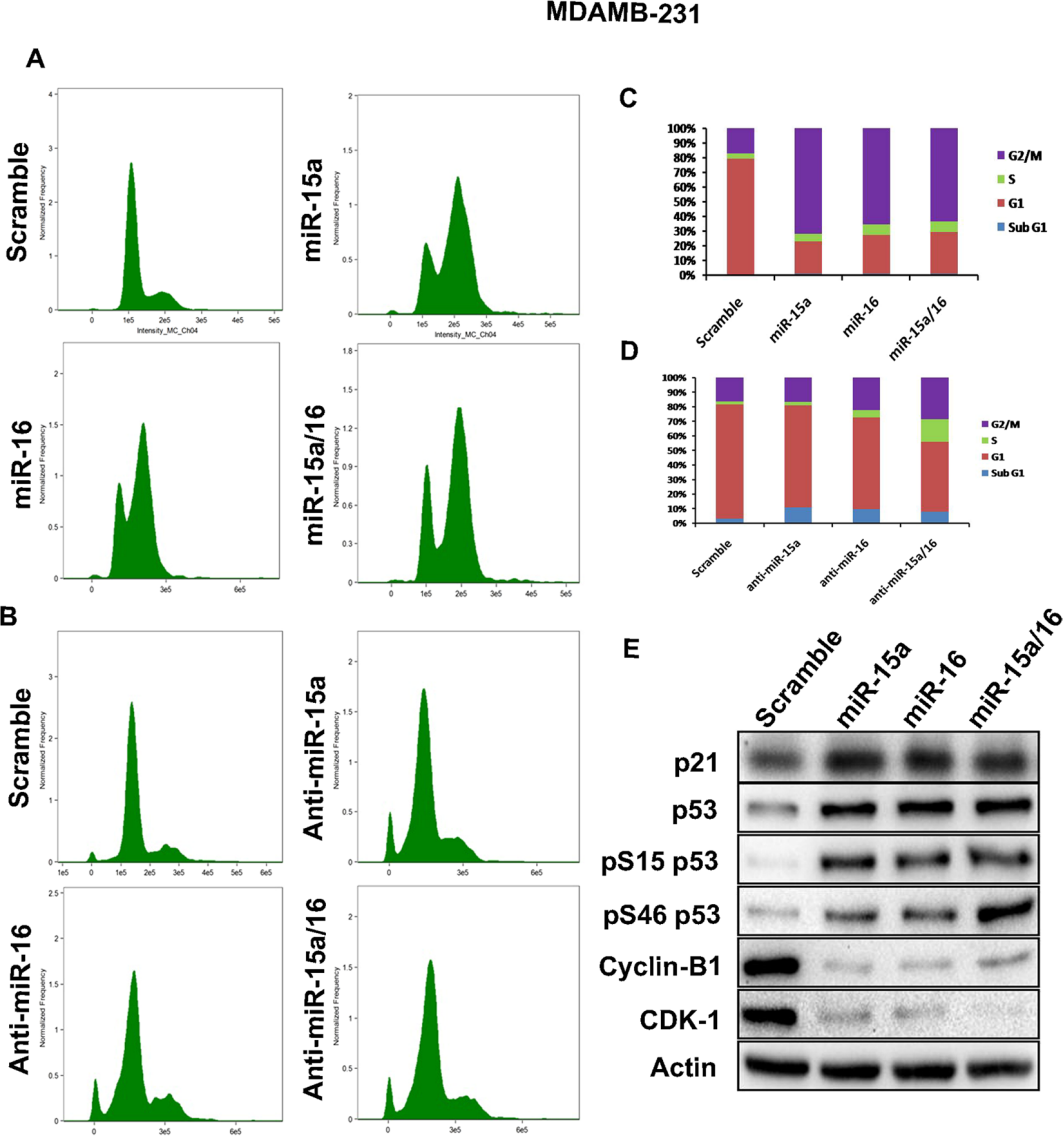
miR-15a/16 mediated of G2/M checkpoints activation. miR-15a, miR-16 and both miR-15a/16 were overexpressed in MDAMB-231 cells and cell cycle analysis was performed. Scramble miRNA vector used as a control (**A**). Anti-miR-15a, anti-miR-16 and both anti-miR-15a/16 were overexpressed in MDAMB-231 cells and cell cycle analysis was performed. Scramble miRNA vector used as a control (**B**). Graph bars represent the percentage of cells in each cell cycle phase (**C,D**). In overexpressed miR-15a, miR-16 and miR-15a/16 cells p21, p53, p53(S46), p53(S15), CDK1, Cyclin-B1 protein expression levels were checked. Actin served as gel loading control. Blots were cropped to enhance the representation. Scramble miRNA vector was used as transfection control (**E**).

Ectopic expression of BMI1 reduces etoposide-induced G2/M arrest and the knock-down of BMI1 sensitizes cells to G2/M arrest^50^. As miR-15a, miR-16 significantly down-regulateBMI1 we wanted to check the effect of miR-15a, miR-16 expression with regard to G2/M checkpoints activation. MDAMB-231 cells were transfected withmiR-15a, miR-16, both miR-15a/16 as well as with anti-miR-15a, anti-miR-16 or both, following which cell cycle analysis was performed by flow cytometry. Scramble miRNA vector transfected cells served as a control. Overexpression of miR-15a, miR-16 or both miR-15a/16 increased cell accumulation in the G2/M cell cycle phase as compared to control cells (Fig. 6A). Cells transfected with anti-miRs did not show any significant cell cycle alterationswhen compared to control cells (Fig. 6B). The percentage of cells in each cell cycle phase are presented in a quantitative graphical form (Fig. 6C,D). Next, we wanted to examinethe expression of proteins involved in G2/M checkpoint activation upon overexpression of these miRNAs. Ectopic expression of miR-15a, miR-16 and both lead to down-regulation of CDK-1, Cyclin-B1 that play a crucial role in G2/M progression. On the other hand phospo-p53(S46), phospo-p53(S15), p53, and p21 were up-regulated (Fig. 6E). These results lead us to concludethat miR-15a, miR-16 overexpression induced G2/M checkpoint activation via BMI1.

### Overexpression of BMI1 and inhibition of endogenous miR-15a/16 strongly antagonizes miR-15a/16 mediated BMI1/Ub-H2A DNA damage repair axis

**Figure 7:**
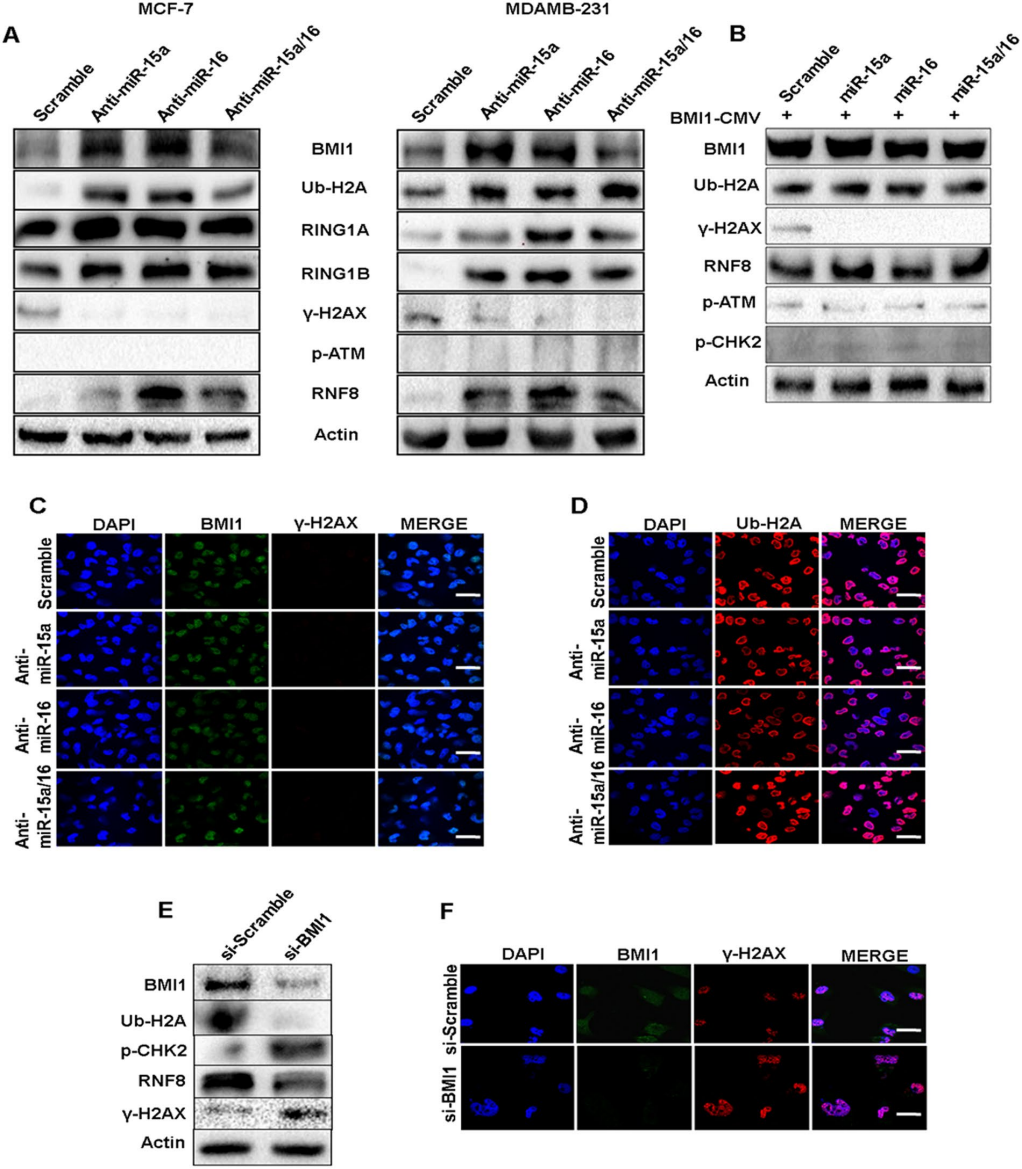
Rescue of BMI1 level as well as inhibiting endogenous miR-15a, miR-16 reduces miR-15a, miR-16 mediated DNA damage. Expression of BMI1, ϒ-H2AX, p-ATM, Ub-H2A and RNF8 were checked in MCF-7, MDAMB-231 cells transfected with anti-miR-15a, anti-miR-16 or both anti-miR-15a/16. Actin served as gel loading control. Blots were cropped to enhance the representation (**A**). Expression of BMI1, ϒ-H2AX, Ub-H2A, RNF8, p-CHK2, p-ATM were checked in MDAMB-231 cells co-transfected with miR-15a, miR-16, miR-15a/16 and pT3-EF1a-Bmi1 plasmid that lack 3′UTR. Scramble miRNA vector used to serve as a control. Actin is used as a gel loading control. Blots were cropped to enhance the representation (**B**). Immunofluorescence studies showing accumulation of BMI1, ϒ-H2AX (**C**), Ub-H2A (**D**) in MDAMB-231 cells transfected with anti-miR-15a anti-miR-16 or both anti-miR-15a/16 under etoposide- treated conditions. Bar indicates 200 μm. Expression of BMI1, Ub-H2A, γ-H2AX, p-CHK2, RNF8 were checked in MDAMB-231 cells transfected with either scrambled si-RNA or si-RNA specifically against BMI1. Actin is used as a gel loading control. Blots were cropped to enhance the representation (**E**). Immunofluorescence studies showing the localization of BMI1 and γ-H2AX foci in cells treated with scramble si-RNA (si-scrambled) or si-RNA against BMI1 (si-BMI1). Cells show an increase in γ-H2AX foci upon depletion of BMI1. Bar indicates 200 μm (**F**).

To confirm that miR-15a and miR-16 down-regulate BMI1 upon etoposide-induced DNA damage, anti-miR-15a, anti-miR-16 and both anti-miR-15a/16 were transfected to MCF-7 and MDAMB-231 cells. 36 hrs post-transfection cells were treated with etoposide for 2 hrs and were allowed to repair for 8 hrs. When total protein isolated from these cells was probed withBMI1 antibody,significant up-regulation of BMI1 level was seen in both MCF-7 and MDAMB-231 cells (Fig. 7A). We further extended these experiments to see the effect on proteins that are known to be involved in BMI1 dependent DNA damage and repair (Ub-H2A, RING1A, RING1B, p-ATM, γ-H2AX, and RNF8). After anti-mir-15a, anti-miR-16 or both anti-miR-15a/16 transfection and subsequentetoposide-induced DNA damage, γ-H2AX expressionwas decreased while RING1A, RING1B, and RNF8 expressionwere increased. The level of H2A-K119ub was also increased but no change inp-ATM was detected in either MCF-7 and MDAMB-231 cells (Fig. 7A). Immunofluorescence results showed no change in the expression of BMI1,γ-H2AX, Ub-H2A in MDAMB-231 cells transfected with anti-miRs compared to control cells (Fig. 7C,D).

Further to prove whether recovery of BMI1 levels in the presence of overexpressed miR-15a, miR-16, or both miR-15a/16 can revert the observed phenomena, we overexpressed BMI1in pT3-EF1a-BMI1^51^, which is lacking BMI1 3′UTR, in MDAMB-231 cells and co-transfected it with miR-15a, miR-16 and both miR-15a/16 followed by treatment with etoposide for 2 hrs and allowed to repair for 8 hrs. Total protein was isolated and BMI1 protein level was evaluated. An equal expression of BMI1 under all the conditions was observed. Results showed a decrease in γ-H2AX with no significant change in the expression of, p-CHK2 and p-ATM. The expression of RNF8 and level H2A-K116ub was moderately increased in cells co-transfected with overexpressed BMI1 and miR-15a, miR-16 and miR-15a/16 when compared with cells co-transfected with scrambled miRNA vector and pT3-EF1a-BMI1vector (Fig. 7B).

This shows that the maintenance of the levels of BMI1 is very crucial in DNA damage repair, as down-regulation of it, via overexpression of miR-15a and miR-16 induces the cells to undergo irreversible DNA damage followed by apoptosis. On the contrary, cells can be protectedfrom DNA damage and cell death by either increasing BMI1 protein levels or by masking the endogenous miRNAs by means of anti-miRs.

### Silencing of BMI1 in cells with chemically induced DNA damage confirms the role of miR-15a miR-16 in DNA damage and repair

To validate the role of miR-15a, miR-16 in etoposide-induced DNA damage via down-regulation of BMI1, we first used si-RNA to knockdown BMI1. We also transfected cells with si-Scramble that served as a control. 36 hrs post-transfection, cells were treated with etoposide for 2 hrs and allowed to repair for 8 hrs. Total protein from the same cells was isolated and probed with BMI1 antibody. There was a clear decrease in the level of BMI1 expression upon si-BMI1 transfection when compared with control. There was also a decrease in the protein expression of RNF8 and levels of ub-H2A at 116 whereas the expression of γ-H2AX and p-CHK2 were increased in si-BMI1 transfected cells (Fig. 7E).

Cells were also visualized under the confocal microscope to detect the intensity of damage that persisted over time when treated with both si-Scramble and si-BMI1. Cells were grown on coverslips and immunofluorescence performed with anti γ-H2AX antibody. Depletion of BMI1 increased DNA damage foci (γ-H2AX) in the nucleus when compared to cells having normal levels of BMI1 (Fig. 7F). This clearly proved that cellular BMI1 levelscan protect againstgenotoxic stress, DNA damage and promote cell survivability.

### miR-15a/16 sensitizes breast cancer cells to the chemotherapeutic drug Doxorubicin

**Figure 8:**
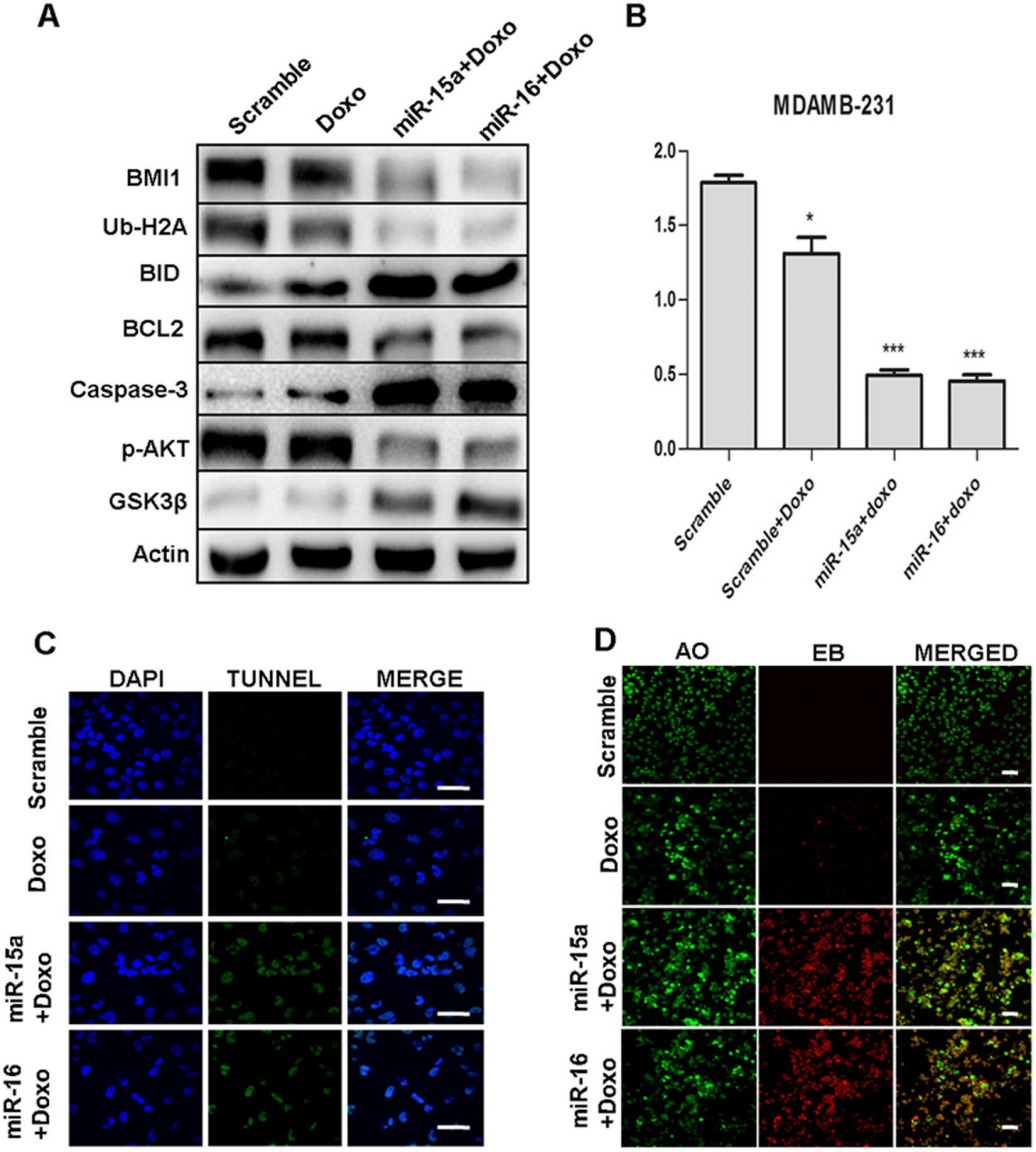
miR-15a/16 sensitizes the breast cancer cells to the chemotherapeutic drug Protein expression of BMI1, Ub-H2A, BID, BCL2, Caspase-3, p-AKT, GSK3β were checked in MDAMB-231 cells ectopically expressed with miR-15a, miR-16 or both miR-15a/16 and treated with doxorubicin. Actin served as gel loading control. Scramble miRNA vector was used as transfection control. Blots were cropped to enhance the representation (**A**). Results of MTT assay showing the effect of miR-15a, miR-16 treated with doxorubicin in MDAMB-231 cells. Scramble miRNA vector was used as control. Error bars indicate ± S.E. of n = 3 (**B**). Tunel assay showing the apoptotic cascade of miR-15a, miR-16 ectopically expressed in MDAMB-231 cells treated with doxorubicin. Scramble miRNA vector was used as control. Bar indicates 200 μm (**C**). Acridine orange/Ethidium bromide double staining represents the live (Green), early apoptotic (Yellow) and late apoptotic/dead (orange/red) cells of miR-15a, miR-16 ectopically expressed in MDAMB-231 cells treated with doxorubicin. Bar indicates 100 μm (**D**).

Doxorubicin is an important chemotherapeutic agent against breast cancer. Silencing of BMI1 expression renders breast cancer cells more sensitive to doxorubicin and induces apoptosis^15^. Here we wanted to check whether the ectopic expression of miR-15a, miR-16 sensitizes breast cancer cells to doxorubicin. Cells were made to overexpressed miR-15a, miR-16 and were treated with 1 µg/ml doxorubicin and allowed to incubate for 48 hrs after which the expression of the proteins involved in apoptosis and survival were checked. Scramble miRNA vector was used as a control. Upon transfection with miR-15a, and miR-16 along with the treatment of doxorubicin, cells showed significant down-regulation of BMI1, Ub-H2A compared to scramble and control cells treated with doxorubicin. Similarly, the pro-apoptotic proteins like BID, BAX, Caspase-3 expression showed elevated expression. The expression of survival protein p-AKT was also down-regulated and GSK3β was up-regulated in the same manner (Fig. 8A). It is already reported that miRNAs play role in chemoresistance in cancers^52^. Whether miR-15a, miR-16 affect the sensitivity of breast cancer cells to the chemotherapeutic drug is not fully understood. To determine this we ectopically expressed miR-15a, miR-16 to breast cancer cells and treated with 1 µM of doxorubicin for 24 hrs following which MTT assay was performed to measure the cytotoxicity levels. Overexpressed miR-15a, miR-16 showed a reduction in proliferation rate with increased cytotoxicity (Fig. 8B). Further to confirm the precise mechanism by which up-regulation of miR-15a, miR-16 enhance chemosensitivity and the apoptotic cascade of breast cancer cells we performed Tunel assay, which is a common method for detecting DNA fragmentation that results in apoptotic signalling cascades. Results showed that the apoptotic rate of ectopically expressed miR-15a, miR-16 in doxorubicin-treated cells were significantly higher (Fig. 8C). Further, in order to differentiate the live and apoptotic cells, upon overexpression of miR-15a, and miR-16 with doxorubicin treatment we performed Acridine orange/Ethidium bromide (AO/EB) double staining. This method detects viable (live) and nonviable (apoptotic) cells. Acridine orange enters both live and apoptotic cells and emits green fluorescence when bound to double-stranded nucleic acid (DNA) or red fluorescence when bound to single-stranded nucleic acid (RNA), while ethidium bromide exclusively stains death cells by binding to DNA where the membrane integrity is lost and emits red fluorescence. As result miR-15a, and miR-16 overexpression with doxorubicin treated cells showed increased percentage of apoptosis compared to control and only doxorubicin treated cells (Fig. 8D). These studies, therefore, conclude that up-regulation of miR-15a, miR-16 sensitizes breast cancer to chemotherapeutic drug doxorubicin by targeting BMI1 and this approach might be the potential strategy for the treatment of breast cancer.

## Discussion

BMI1 is known for its oncogenic properties in various cancers and is considered as a prognostic marker for breast cancer^2,10,11^. Silencing BMI1 significantly inhibits tumorogenesis in various cancers including breast cancer. BMI1 is part of the E3 ubiquitin ligase complex that plays a crucial role in DNA damage and repair by mono-ubiquitinating H2A at lysine (K) 119 (H2A-K119Ub), which facilitates DNA repair and maintains genome integrity. The other PRC1 group of polycomb proteins (RING1A, RING1B) are also involved in DDR by mono-ubiquitinating H2A-K119Ub. BMI1 recruitment to the damaged site is mediated by phosphorylation of ATM, ATR, H2AX and RNF8^20,21^. Due to its oncogenic role as well as its function in the control of DNA damage response and repair, BMI1 stands as a very attractive target for breast cancer therapeutics.

Disturbance in the post-transcriptional regulation of BMI1 disrupts the homeostasis in the DNA repair mechanism by impairing ubiquitin conjugation, a highly essential initial step for the subsequent recruitment of many DNA damage response proteins. We observed in our study that miR-15a and miR-16 binds strongly to the 3′UTR of BMI1 and inhibits BMI1 protein synthesis. miR-15a and miR-16 are located at chromosome location 13q14.3, which is frequently deleted or down-regulated in CLL^53^. In this study, we have shown that both miR-15a and miR-16 serve as major post- transcriptional regulators of BMI1 and suppresses the expression level of BMI1, and also, other polycomb group proteins like RING1A, RING1B, EZH2, MEL18 in etoposide-induced DNA damage response (Figs 3 and 4). Ectopic expression of miR-15a, miR-16 and both miR-15a/16 significantly abolished DNA damage mediated induction of BMI1and levels of ubiquitination of H2A at 119 positions in BMI1 dependent ubiquitination pathway (Figs 3 and 4). We also observed that ectopic expression of miR-15a, miR-16 and both miR-15a/16 produced higher residual DNA damage when compared to scramble miRNA vector, under etoposide-induced DNA damage conditions, as confirmed by comet assay (Fig. 2A), whereas, transfection of anti-miR-15a, anti-miR-16 and both did not induce DNA damage (comet) (Fig. 2B). It also contributed to the induction of γ-H2AX protein which is a marker for DNA double-strandbreak (Figs 3 and 4). Interestingly expression of RNF8, RNF168, BRCA1protein was down-regulated whereas expression of p-CHK2, p-ATM was up-regulated in etoposide-treated cells having overexpressed miRNAs (Figs 3B and 4B). Immunofluorescence studies clearly, showed an increase in γ-H2AX foci (Figs 3C and 4C) as well as localization of p-CHK2 (Fig. S3B,D), p53BP1 (Figs 3F and 4F) to the damage sites in both MCF-7 and MDAMB-231 cells. Clearly,there was a drastic decrease in the localization of BMI1 (Figs 3C, 4C), Ub-H2A (Figs 3E, 4E), RING1A (Fig. S3A,C), and RING1B (Figs 3D, 4D) to the sites of damage. This confirmed that miR-15a and miR-16 mediated the regulation of BMI1 post-transcriptionally. Overexpression of miR-15a, miR-16 drastically down-regulates BMI1 mRNA expression compared to other genes involved in DNA damage response (Fig. 5B,C). This is due to the fact that miR-15a/16 have the binding site at 3′UTR of BMI1 which regulates its expression. BMI1leads to DNA double-stranded break and repair by homologous recombination^21^. Overexpression of miR-15a/16 significantly reduced HR-efficiency (Fig. 5F). We examined cell cycle specific chromosome damage at metaphase to determine whether overexpression of miR-15a/miR-16, and BMI1 has an impact on chromosome break repair. No significant difference in G1- or G-2 specific aberrations were found in cells with and without overexpression of miR-15a/miR-16, and BMI1, however, an increased number of S-phase specific aberrations (radials) was observed in cells expressing miR-15a/miR-16, suggesting that depletion of BMI1 does effect the HR mediated repair as indicated by increased number of S-phase specific chromosomal aberrations (Fig. 5D,E). Consistent with the increased number of S-phase aberrations, we found that cells overexpressing miR-15a/miR-16 has reduced HR mediated DSB repair, whereas cells overexpressing BMI1 have slightly increased frequency of HR mediated DSB repair (Fig. 5F).

Literature suggests that miR-15a/16 and miR-15b/16 regulate cell cycle and apoptosis by targeting CDK6, Cyclin D3 (CCND3), and Cyclin E1 (CCNE1)^54,55^. It is also reported that knock-down of BMI1 sensitized cells to G2/M cell cycle arrest^50^. We, therefore, wanted to check the expression of miR-15a, miR-16 in cell cycle regulation. Overexpression of miR-15a, miR-16 and both miR-15a/16 facilitated accumulation of cells at G2/M cell cycle phase when compared to scramble miRNA (Fig. 6A) and ectopic expression of anti-miR-15a, anti-miR-16 did not accumulate the cells in G2/M cell cycle phase (Fig. 6B). The same conditions increased the protein expression of p21, p53, phospo-p53 (S15), phospo-p53 (S46) and decreased the CDK1, Cyclin-B1 which is involved in G2/M phase activation (Fig. 6E).

In order to confirm the hypothesis, we further cross checked whether the events of DNA damage repair regained their levels by means of BMI1 overexpression along with miR-15a, miR-16 and both miR-15a/16 overexpression upon etoposide treatment. Results were supportive to our hypothesis as the gain in the BMI levels in etoposide-induced DNA damage under the higher levels of mir-15a and miR-16 and both miR-15a/16, over a time period, regained the cell’s ability to bring back the normal levels of ubiquitinatedH2A and survival protein Bcl-2 (Fig. 7B). On the other hand loss of endogenous levels of miR-15a and miR-16 showed similar phenomenon of regain in the repair response, where BMI1, RING1A, RING1B, Ub-H2A levels were elevated and γ-H2AX levels decreased as compared to cells having endogenous expression of miR-15a and miR-16 (Fig. 7A). We also proved that knock-down of BMI1 levels decreased ubiquitination of H2A at 119 positions and increased γ-H2AX and p-CHK2 levels in etoposide-induced DNA damage (Fig. 7E). This clearly proves that BMI1 plays very crucial role in maintaining the genome integrity by responding to the DNA damage and initiating repair through ubiquitination of H2A which in turn facilitates the recruitment of various DNA damage response proteins to the damage foci for initiating repair. Depletion of endogenous miR-15a and miR-16 or overexpression of BMI1 in MCF-7 and MDAMB-231 cells regained their DNA repair, whereas depletion of BMI1 by means of si-RNA could not regain repair and the cells underwent irreversible damage followed by apoptosis. This data can help in designing better therapeutics against various cancer types in which BMI1 is overexpressed. In most of the cancer, BMI1 is associated with tumorogenesis and overexpression of BMI1 confers drug resistance^56^. Silencing of BMI1 sensitizes the human cancers to chemotherapeutic drug^14,15^. Reports suggest that miRNAs could able to sensitized cancers cells to chemotherapeutic drug^52,57,58^. We hypothesized that miR-15a/16 could sensitize the breast cancer cells to the chemotherapeutic drug as our results show that miR-15a/16 drastically decreased the BMI1 expression in breast cancer cells. Doxorubicin was taken as it is a well-known drug for breast cancer but it is often associated with severe toxicity. Ectopic expression of miR-15a, miR-16 sensitizes breast cancer cells to doxorubicin by increasing doxorubicin-induced apoptosis as confirmed by performing MTT, Tunel assay,Acridine orange and Ethidium bromide (AO/EB) staining (Fig. 8B,C,D). Western blots show BMI1, Ub-H2A, pro-apoptotic and anti-apoptotic and survival protein expression (Fig. 8A). Homologous recombination (HR) repair increases cell cycle progression by modulating the expression of cyclin-dependent kinases (CDKs)^59^. In the cell cycle HR occurs during the S and G2 and is therefore more significant in cancer cells when compared to healthy cells^60^. Persistence of DDR controls HR which is organized by multiple kinase and ubiquitin ligases that directly coordinate a cell cycle arrest and induces apoptosis^59^. Here we emphasized the unique role of miR-15a/miR-16 cluster in their dramatic participation in breast cancer by reducing the oncogene and DNA repair protein BMI1, and therefore inducing impaired repair of damaged DNA via homologous recombination and apoptosis. Additionally, miR-15a, miR-16 sensitizes the breast cancer to the chemotherapeutic drug doxorubicin. miR-15a, miR-16 are hence potentially, a new class of cancer biomarkers which can regulate BMI1 expression and hence targeting them can be a better therapeutic approach for human cancer incoming future.

## Material and Method

### Target analysis

miRNA binding sites in BMI1 3′UTR were identified with the miRTarBase (http://mirtarbase.mbc.nctu.edu.tw/) software tool.

### Cell line and Cell culture

MCF-7 and MDAMB-231 cells were obtained from American Type Culture Collection (ATCC, USA). MCF-7 cells were maintained in DMEM and MDAMB-231 cells were maintained in RPMI supplemented with 10% fetal bovine serum (FBS) 100 U/ml, Penicillin and 100 mg/ml streptomycin sulfate (Sigma) at 37 °C with 5% CO2 in a 95% humidified atmosphere.

### Dual Luciferase Assay and Cell transfection

miRNAs and BMI1 3′UTR were co-transfected using lipofectomine-2000 (Invitrogen). Anti-miR-15a and anti-miR-16 were procured from (Exiqon, cat #. has-miR-15a-5p 4100999-001, has-miR-16-5p 4102068-001). microRNAs miR-15a (cat #. PMIRH15PA-1) miR-16 (cat #. PMIRH16PA-1) and Scramble miRNA vector (cat #. PMIRH000PA-1) were procured from System Bioscience. To confirm that miR-15a, miR-16 has abinding site in the BMI13′UTRand regulates expression in both MCF-7 and MDAMB-231, cells were seeded in 12-well plates and co-transfected with individual miRNA along with wild (wt) BMI1 3′UTR psiCHECK2 or mutant (Mut) BMI1 3′UTR psiCHECK2 reporter plasmids. 48 hrs after co-transfection, the lysate was prepared as per the protocol. Luciferase assays were performed using the Dual-Luciferase Reporter Assay kit (Promega cat #. E1910). Renilla luciferase activity was normalized to firefly luciferase activity for each sample. Scramble microRNA vector was used as a control. pT3-EF1a-Bmi1 was a gift from Xin Chen (Addgene plasmid # 31783) and overexpressed with miR-15a, miR-16, both miR-15a/16 in MDAMB-231 cells. Cells were allowed to grow for 36 hrs followed by etoposide treatment for 2 hrs and were allowed to repair for 8 more hrs, The lysate was prepared as per the protocol. Western blot was performed to check the expression of BMI1 dependent DNA damage proteins. Scramble microRNA vector was used as a control. Si-BMI1 was procured from EUROGENTEC (Cat #. 5647715) and transfected in MDAMB-231 cells. Cells were allowed to grow for 36 hrs followed by etoposide treatment for 2 hrs and were allowed to repair for 8 more hrs, followed by lysate was prepared as per the protocol. Western blot was performed to check the expression of BMI1 dependent DNA damage proteins.

### MTT

Cytotoxicity level was checked by performing MTT assay. MDAMB-231cells (15 × 10^3^) were seeded in 96 well plates and allowed to grow. After 24 hrs, miR-15a, miR-16 were ectopically expressed and incubated for 24 hrs. Scramble microRNA vector was used as a control. Thereafter the cells were treated with 1 µM of doxorubicin followed by further incubation for 24 hrs. MTT reagent was added according to manufacturer’s instruction (Molecular Probes, Vybrant MTT Cell Proliferation Assay Kit, #V13154) and cell viability was determined by taking optical density values at 570 nm (Thermo Scientific Varioskan Flash). Each experiment was repeated three times and the mean average was plotted.

### Western Blot Analysis

miR-15a, miR-16 and miR-15a/16 as well as anti-miR-15a, anti-miR-16, or combined anti-miR-15a/16 were transfected to MCF-7, MDAMB-231cells in a 60 mm dish and incubated for 36 hrs. Thereafter it was treated with 5 µM of etoposide for 2 hrs to produce DNA damage. Media was changed and the cells were allowed to repair for 8 hrs under normal conditions. Cell lysates were prepared with RIPA lysis buffer (Sigma cat # R0278). Scramble microRNA vector was used as a control. Equal amount of proteins were loaded and separated by SDS-PAGE and transferred onto PVDF membrane. Blots were hybridized with antibodies against anti-rabbit-BMI1 (Cell Signaling cat #. 6964s 1:1000 dilution), anti-rabbit-RING1A (Cell Signaling cat #. 13069 1:1000 dilution), anti-rabbit-RING1B (Cell Signaling cat #. 5694 1:1000 dilution), anti-rabbit-EZH2 (Cell Signaling cat #. 5246 1:1000 dilution, anti-rabbit-γ-H2AX (Cell Signaling cat #. 2577s 1:1000 dilution), anti-mouse p-ATM (Calbiochem cat #. DR1002 1:2000 dilution), anti-rabbit p-CHK2 (Cell Signaling cat #. 2666s 1:1000 dilution), anti-rabbit Ub-H2A (Cell Signaling cat #. 8240s 1:1000 dilution), anti-rabbit RNF8 (Abcam cat #. 105362 1:1000 dilution), anti-rabbit-RNF168 (Novus Biological cat #. NBP1-76324PEP 1:1000 dilution), anti-mouse-MEL16 (SDIX cat #. G2910-331A02 1:1000 dilution), anti-rabbit p21 (Cell Signaling cat #. 2947s 1:1000 dilution), anti-rabbit BID (Cell Signaling cat #. 20025 1:1000 dilution), anti-rabbit BCL2 (Abcam cat #. 32124 1:1000 dilution), anti-mouse Caspase-3 (Imgenex cat #. IMG-144A 1:500 dilution),anti-rabbit-p53BP1 (Cell Signaling cat #. 4939 1:1000 dilution), anti-rabbit-CyclinB1(Abbiotec cat #. 251217 1:500 dilution) anti-rabbit-CDK1(Abbiotec cat #. 251784 1:500 dilution), anti-rabbit-GSK3β (Cell Signaling cat #. 93155 1:1000 dilution), anti-rabbit-pAKT (Cell Signaling cat #. 40602 1:1000 dilution),anti-rabbit-BRCA1 (Millipore cat #. 07-434), anti-rabbit-phospo-p53 (ser46) (Cell Signaling cat #. 2521 1:1000 dilution), anti-rabbit-phospo-p53 (ser15) (Cell Signaling cat #. 9284S 1:1000 dilution) anti-mouse Actin (Abcam cat #. ab8224 1:1000 dilution), and anti-rabbit p53 (Cell Signaling cat #. 2527s 1:1000 dilution). Goat anti-rabbit (IgG-HRP Santa Cruz cat #. sc-2004) and goat anti-mouse (IgG-HRP Santa Cruz cat #. sc-2005) were used as Secondary antibodies.

### Immunoblot Analysis

For immunoblotting 2 x 10^5^ MCF-7 and MDAMB-231 cells were seeded on the sterile cover slip. After 24 hrs, cells were transfected with miR-15a, miR-16, and both miR-15a/16. Scramble microRNA vector was used as control. 36 hrs post-transfection, 5 µM etoposide was added and allowed to induce DNA damage for 2 hrs followed by 8 hrs of repair phase. Cells were fixed using 4% paraformaldehyde followed by three washes of 5 minutes each in PBST (Phosphate Buffered Saline with Tween® 20). Slides were further incubated separately with following primary antibodies: anti-rabbit-γ-H2AX (Cell Signaling cat #. 2577s 1:1000 dilution), anti- mouse BMI1(Millipore cat #. 05-637), anti-rabbit-RING1A (Cell Signaling cat #. 13069 1:1000 dilution), anti-rabbit-RING1B (Cell Signaling cat #. 5694 1:1000 dilution), anti-rabbit Ub-H2A (Cell Signaling cat #. 8240s 1:1000 dilution),anti-rabbit p-CHK2 (Cell Signaling cat #. 2666s 1:1000 dilution) and anti-rabbit-p53BP1 (Cell Signaling cat #. 4939) overnight at 4 °C. PBST washes were repeated for three times of 5 minutes interval each. Slides were then incubated with the anti-mouse-FITC anti-rabbit-CY3 secondary antibody (Jackson Immuno Research). Slides were finally washed in PBS, dried and mounted with Vectashield mounting media containing DAPI that counter stains the nucleus (Invitrogen, cat. # D21490). Slides were viewed under confocal the microscope (Olympus Model no FV1000).

### RNA Isolation and RT-PCR

Total RNA was isolated using TRIzol reagent (Invitrogen, USA) according to the manufacturer’s instructions. Concentrations were determined at an absorbance of 260 nm. cDNA was synthesized (1 μg for mRNA) using Ecodry (Clonetech, USA) and quantified using Nanodrop (Thermo Scientific USA, ND-0859) at 260/280 ratio. mRNA levels were determined by performing qRT-PCR using primer sets for BMI1, RING1A, RING1B, PHC1, PHC2, CHK1, CHK2, ATM, ATR, DNA-PK, KU-70, KU-80, RAD50, RAD51,BRCA1, p53, p53BP and GAPDH. The primers sequences are provided in the supplementary section (Table S1). GAPDH was used as an endogenous loading control. qRT- PCR was performed using Applied Biosystem Power SYBR Green PCR Master Mix in 7900HT Fast Real-Time PCR System (Applied Biosystem, USA). The reaction mixture was prepared for a reaction volume of 10 μl containing: 3 μl PCR-H2O, 1 μl forward primer (0.5 μM), 1 μl reverse primer (0.5 μM), 5 μl Power SYBR® Green PCR Master Mix and 1 μl cDNA (50 ng) as a template. Following amplification protocol, quantification procedures were carried out- Hot Start (50 °C for 2 min), initial denaturation (95 °C for 10 min), denaturation (95°c for 15 sec) annealing and extension (60 °C for 1 min) program repeated for 40cycles with each step under fluorescence measurement mode. ΔΔCt method was used to analyze the qRT-PCR data.

### Chromosome aberration analysis

Cell cycle stage-specific chromosome aberrations were analyzed at metaphase after irradiation as previously described (Singh M *et al*.^47^). For G1-phase chromosomeaberration analysis, cells were exposed to 3 Gy then incubated for 12 h and metaphase cells were as described previously. For S-phase specificchromosome aberrations analysis, exponentially growing cells were pulse labeled with BrdU, irradiated with 2 Gy, incubated for 5 hr and metaphases were analysed by the previously describedprocedure. For G2-phase specific aberrations, cells were 1 Gy irradiated andthose in metaphase were collected 1 h after treatment.

G1-phase specific chromosome aberrations includedicentrics, centric rings, interstitial deletions/acentric rings and terminaldeletions. S-phase chromosome aberrations includetriradialandquadriradial exchanges per metaphase. G2-phase chromosome aberrationswere measured by counting chromatid breaks and gaps permetaphase, as previously described (Pandita RK *et al*.^61^).

### Comet Assay

MCF-7 and MDAMB-231 cells were transfected with miR-15a, miR-16, and both miR-15a/16 and incubated at 37 °C for 36 hrs. Cells were then treated with 5 µM of etoposide for 2 hrs and allowed to repair for 8 hrs followed by the trypsinization of the cells. Cells were then put in low melting agarose and allowed to spread on the glass slide. It was further incubated in lysine solution (2.5M NaCl 146.1 g, 100 mM EDTA 37.2 g, 10 mMTrizma base 1.2 g, fresh prepared 1% Triton X-100, and 10% DMSO) and kept under refrigerated condition for 30 min. The slides were further incubated in alkaline lysis buffer (2.5 M NaCl, 100 mM EDTA, 10 mMTrizma base) for 20-30 min and subjected to electrophoresis at 24 V and 300 mA for 30 min. Slides were washed with neutralization buffer and stained with cyber green (1 mg/mL). Images were captured using confocal microscope (Olympus, Model No. FV1000.) Scramble microRNA vector was used as a control.

### Homologous Recombination Assay

The impact of miR-15a, miR-16 and BMI1 overexpression on DNA DSB repair by Homologous recombination assay (HR) was determined by using I-Scel induced DNA DSB in green fluorescent protein (GFP) reconstitution assays. Induction of a site specific DSB was performed as described previously (Gupta A *et al*.^49^).

### Terminal Transferase UTP Nick End Labeling (TUNEL) Assay

Cells were seeded in 6 well plate on coverslips. miR-15a, miR-16 were ectopically expressed in the cells and they were treated with 1 µM doxorubicin for 48 hrs. Further, the cells were washed with DPBS and DNA fragmentation was detected using *In situ* Apoptosis Detection Kit (Tunel) Cat #. MK500 TAKARA BIO INC which specifically labeled 3′-hydroxyl termini of DNA strand breaks using fluorescein isothiocyanate (FITC)-conjugated dUTP. DNA was also labeled with FITC DNA binding dye for 5 min. Images were taken under confocal microscopy (Olympus Model no FV1000).

### Dual (AO/EB) staining to detect apoptosis

Acridine orange and Ethidium bromide (AO/EB) double staining method was performed to detect live (viable) and apoptotic (nonviable) cells. miR-15a, miR-16 were ectopically expressed in the cells and they were treated with 1 µM doxorubicin for 48 hrs. Further, the cells were washed with DPBS and 50 µL of AO/ EB dye mixture (10 µL/mg AO and 10 µL/mg EB in distilled water) in 1:1 ratio was added to each well and incubated for 30 minutes. After incubation, the images were taken under confocal microscope (Olympus Model no FV1000).

### Statistical Analysis

Results are given as Means of three independent experiments ± S.D., employing Students t-test, performed with triplicate values. Values at p < 0.05 were considered as significant.

## Electronic supplementary material

Supplimentary File
