# Erratum: miR-15a/miR-16 down-regulates BMI1, impacting Ub-H2A mediated DNA repair and breast cancer cell sensitivity to doxorubicin

**DOI:** 10.1038/s41598-017-12314-6

**Published:** 2017-10-10

**Authors:** Nibedita Patel, Koteswara Rao Garikapati, Raj K. Pandita, Dharmendra Kumar Singh, Tej K. Pandita, Utpal Bhadra, Manika Pal Bhadra

**Affiliations:** 1 Centre for Chemical Biology, CSIR-Indian Institute of Chemical Technology, Tarnaka, Hyderabad, Telangana State 500007 India; 2 Gene Silencing Group, Centre for Cellular and Molecular Biology, Hyderabad, Telangana State 500007 India; 3 Academy of Scientific and Innovative Research (AcSIR), Training and Development Complex, CSIR Campus, CSIR Road, Taramani, Chennai 600 113 India; 4 Department of Radiation Oncology, Weill Cornell Medical College, The Methodist Hospital Research Institute, Houston, TX 77030 USA

*Scientific Reports* **7**:4263; doi:10.1038/s41598-017-02800-2; Article published online 27 June 2017

The original version of this Article contained a typographical error in the spelling of the author Dharmendra Kumar Singh, which was incorrectly given as Dharmender Kumar Singh.

In addition, the acknowledgements section was incomplete. It now reads:

“N.P. thanks ICMR and G.K.R. thanks UGC for their fellowship. All the authors thank Y. Suresh for the flow cytometry studies. The work is supported by CSIR 12 FYP CSC 0111 and National Institutes of Health grants CA129537 and GM109768 (TKP)”.

These errors have been now been corrected in the HTML and PDF versions of the Article.

