# Supplementary material for: miR-15a/miR-16 down-regulates BMI1, impacting Ub-H2A mediated DNA repair and breast cancer cell sensitivity to doxorubicin
Source: Sci Rep. 2017 Jun 27;7:4263. doi: 10.1038/s41598-017-02800-2 (PMC5487337; doi:10.1038/s41598-017-02800-2)
Supplement: Supplementary file 1 — Supplimentary File [file 41598_2017_2800_MOESM1_ESM.doc]

**Supplementary Data**

**miR-15a/miR-16 down-regulates BMI1, impacting Ub-H2A mediated DNA repair and breast cancer cell sensitivity to doxorubicin**

**Nibedita Patel^1^, Koteswara Rao. Garikapati^1,3^, Raj K. Pandita^4^, Dharmendra Kumar Singh^4^, Tej K. Pandita^4^, Utpal Bhadra^2^ and Manika Pal Bhadra**^1^*

**Fig.S1**

**Etoposide induced cell death in MCF-7 and MDAMB-231 cell lines**


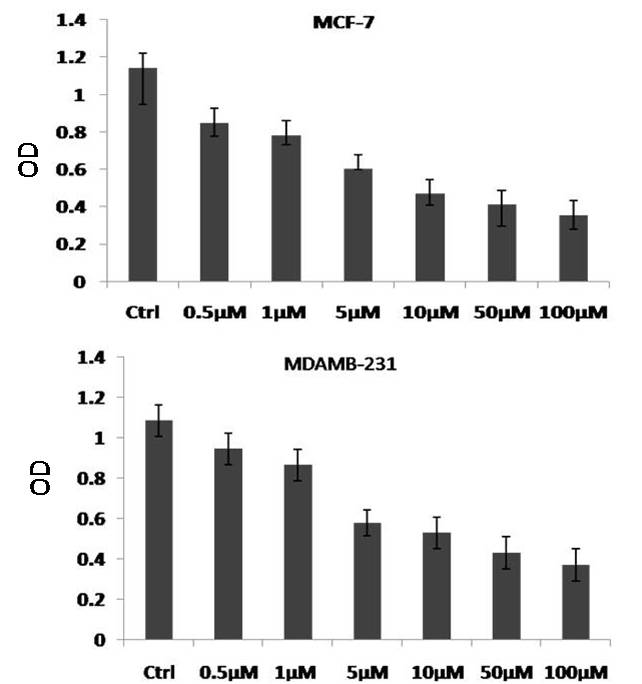


**Fig.S2**

**Immunostaining shows γ-H2AX expression level upon Etoposide treatment**

**
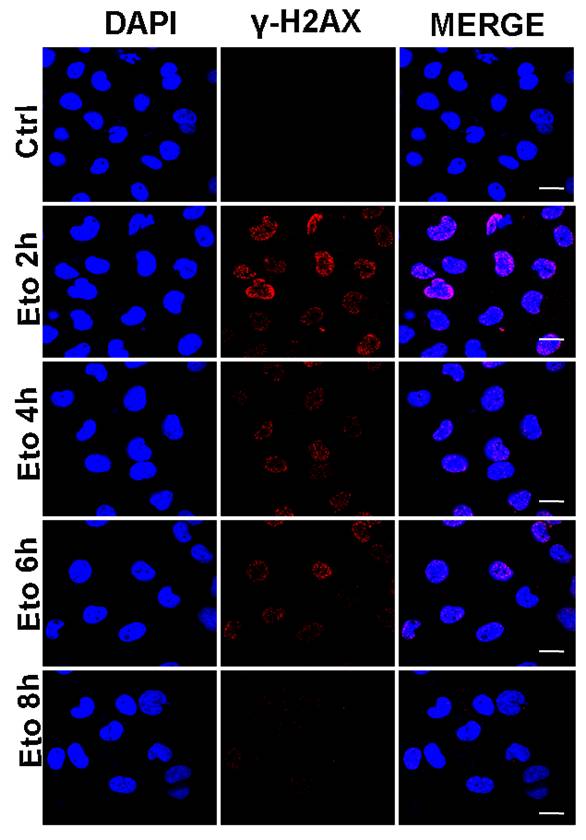
**

**Fig.S3**

**Accumulation of RING1A and pCHK2 in miR-15a/16 overexpressed cells**

**
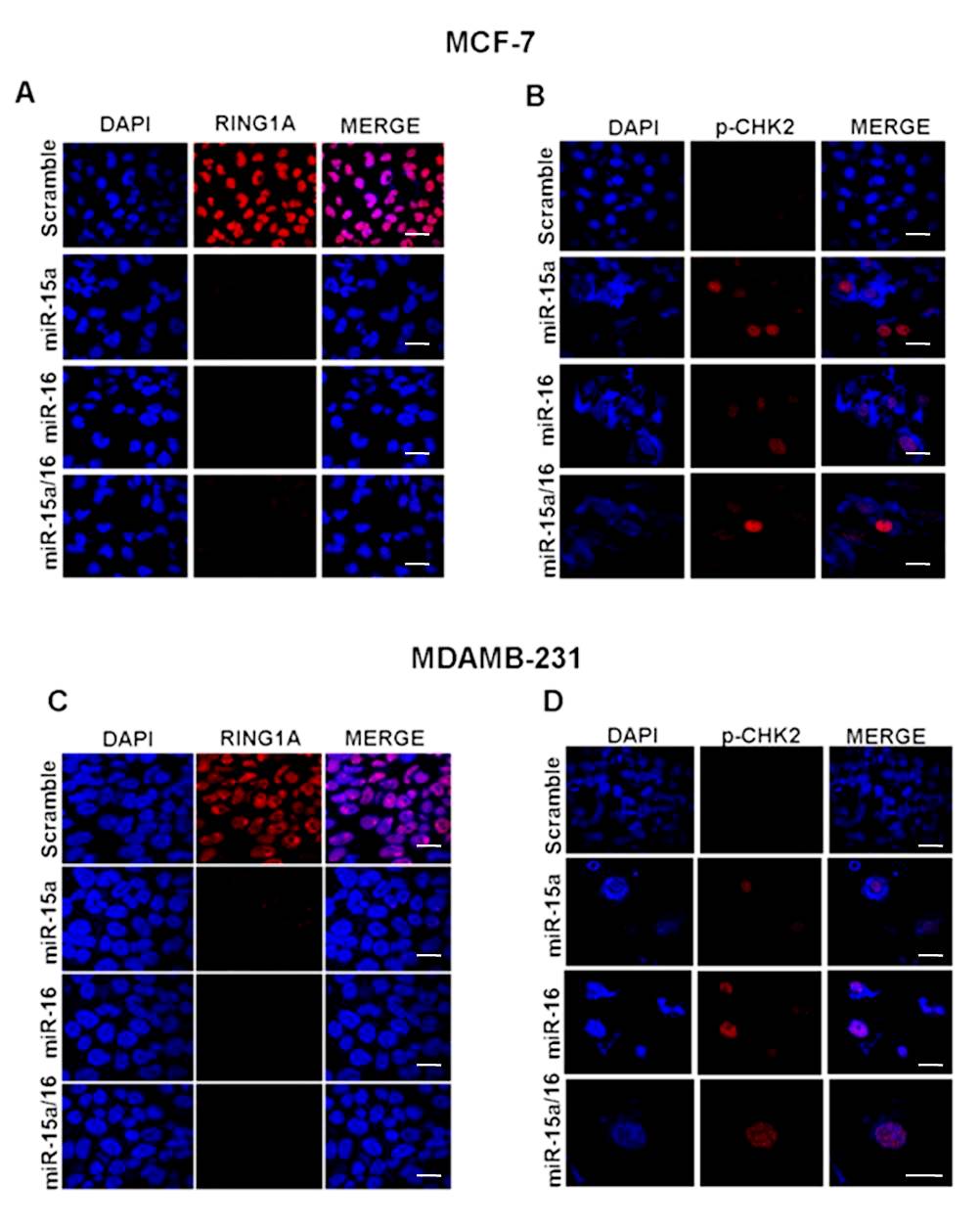
**

**SUPPLEMENTARY FIGURE LEGEND**

**S1: Etoposide induced cell death in MCF-7 and MDAMB-231 cell lines**

MTT assay performed in MCF-7 and MDAMB-231 cells after treatment with 0.5µM to 100µM of etoposide. Treatment of both cell lines with 5µM etoposide showed 50 percentage of cell death. Error Bars represents Standard Error from three independent experiments (Fig.S2A).

**S2:Immunostaining shows γ-H2AX expression level upon etoposide treatment**

5μM of Etoposide was treated and after 2 hrs of incubation media was changed. Further Immunostaining was performed and check the expression of γ-H2AX (DNA damage marker) in different time points like 2hrs, 4hrs, 6hrs, and 8hrs (Fig.S2B).

**S3:Accumulation of RING1A and pCHK2 in miR-15a/16 overexpressed cells** Immuno fluorescence data showing accumulation of RING1A (Fig.S3A, C) and pCHK2 (Fig.S3B, D) in MCF-7 and MDAMB-231 cells transfected with miR-15a miR-16 or both miR-15a/16 under etoposide- treated conditions. Bar indicates 200μm.

**SUPPLEMENTARY TABLE. 1**

Table represent the primers used in the RT-PCR and Cloning/Mutagenesis.

| Sl no | PRIMER | FORWARD 5’ to 3’ | REVERSE 5’ to 3’ |
| --- | --- | --- | --- |
| 1 | GAPDH | GGGAAGGTGAAGGTCGG | TTGAGGTACATGAAGGGGTCA |
| 2 | BMI1 | TTGTTTGCCTAGCCCCAGTA | GAAGAAGTTGCTGATGACCCA |
| 3 | RING1A | AGATGTGAGCTCAGACTCCG | TCACCAGAGTCTTCCGAACC |
| 4 | RING1B | GTTGTATTTCCCGAGCTCCA | TGAGTTCCGTTTGTCTGCAC |
| 5 | PHC1 | ATGAACGACAAGCAGTGCAG | GTGTAGTGCTGGTGTTTGGG |
| 6 | PHC2 | TGCCACCACTTACCAAGGAT | CTGAGATGGGTGACAAGGGT |
| 7 | ATM | GGACAGTGGAGGCACAAAAT | GTGTCGAAGACAGCTGGTGA |
| 8 | ATR | CCGCAAAAGGAGATTTGGTA | TTCGGAAGTGCTGTCATCTG |
| 9 | P53BP | GACGGTACCGAGTGTTAGTCCGGCAGCTT | GACAAGCTTCAGATGGAGGCAACGATACA |
| 10 | P53 | GACGGTACCGAGTCCCGCGGTAATTCTTA | GACAAGCTTGTGTCACCGTCGTGGAAAG |
| 11 | KU70 | AAAAGACTGGGCTCCTTGGT | TGTGGGTCTTCAGCTCCTCT |
| 12 | KU80 | TGCAGCAAGAGATGATGAGG | GAAAGGCAGCTGCACATACA |
| 13 | DNA-PK | CTTTGTCGTGTGGAGGGAAT | CACAACGGGGTTCAGAAGTT |
| 14 | BRCA1 | ACAGCTGTGTGGTGCTTCTGTG | CATTGTCCTCTGTCCAGGCATC |
| 15 | RAD50 | AGCCACAGCAGTTTACTCCC | TGTCGTTCTTTAGGCGCTGT |
| 16 | RAD51 | TGCGACTCGCTGATGAGTTT | CTGGTTGTTGATGCATGGGC |
| 17 | CHK1 | TGTCAGAGTCTCCCAGTGGA | AGGGGCTGGTATCCCATAAG |
| 18 | CHK2 | CAAGGCTCCTCCTCACAGTC | TGGTCCTCAGGTTCTTGGTC |
| 19 | BMI1 3’ UTR | ATTACTCGAGGGAAAAAAATTTTAAACCCCTGA | ATTAGCGGCCGCATAAACAGATACATCTTTCAATGGGC |
| 20 | SDM BMI1 3’UTR 15a | GTCAAATCATTACTTTTACATATATCTTCTGCTTTCTTTAAAAATATAG | CTATATTAAAGAAAGCAGAAGATATATGTAAAAGTAATGATTTAATGAC |
| 21 | SDM BMI1 3’UTR 15a/16 | GTACAGTCCCATTGTAATTCTAATTATAGATGTAAAATG | CATTTTAATCATCTATAATTAGAATTACAATGGGACTGTAC |
